# Prevalence and associated factors of myopia among school children in Bahir Dar city, Northwest Ethiopia, 2019

**DOI:** 10.1371/journal.pone.0248936

**Published:** 2021-03-22

**Authors:** Abel Sinshaw Assem, Mebratu Mulusew Tegegne, Sofonias Addis Fekadu

**Affiliations:** Department of Optometry, School of Medicine, College of Medicine and Health Sciences, University of Gondar, Gondar, Ethiopia; National Eye Institute, UNITED STATES

## Abstract

**Background:**

Myopia is the leading cause of correctable visual impairment and preventable blindness worldwide. Genetic and environmental factors contribute to the development of myopia. Myopia is appearing with greater prevalence in young children.

**Objective:**

This study aimed to assess the prevalence and associated factors of myopia among school children in Bahir Dar city, Northwest Ethiopia, 2019.

**Methods and materials:**

A school-based cross-sectional study was conducted among school children of 6 to 18 years of age in Bahir Dar city from October to November 2019. A pretested interviewer-administered structured questionnaire was used to collect data among 634 participants using a multi-stage sampling technique from primary and secondary schools. Cycloplegic refraction was performed by optometrists for each student with 1% cyclopentolate eye drop, and subjective refraction was carried out to determine the final prescription of the students. Myopia was defined as spherical equivalent refractive error of ≥ 0.5 diopter in either eye. Data were entered into Epi Info version 7 and exported to Statistical Package for Social Sciences version 23 for analysis. Tables, frequency, and mean were used for descriptive statistics. Bivariable and multivariable logistic regression analyses were done to identify risk factors of myopia. Odds ratio with 95% confidence level was determined and variables with p–value of < 0.05 were considered as statistically significant.

**Results:**

Among a total of 601 study participants, 51 (8.49%) were myopic. Age group of 10–13 years (AOR = 6.54: 95% CI = 5.56–10.86), 14–18 years (AOR = 6.32: 95% CI = 5.32–9.69), 2–4 hour per day mobile exposure (AOR = 3.69: 95% CI = 1.63–8.38), > 4 hour per day mobile exposure (AOR = 11.6: 95% CI = 4.41–30.42), near working distance of < 33 centimeter (AOR = 6.89: 95% CI = 2.71–17.56) and outdoor activity (AOR = 3.94: 95% CI = 1.87–8.31) were significantly associated with myopia.

**Conclusions:**

The prevalence of myopia was high among school children in Bahir Dar city. Older age, longer duration of mobile exposure, shorter near working distance were the risk factors for the development of myopia whereas having outdoor activity was the protective factor.

## Introduction

Myopia, commonly known as short-sightedness, is a state of refraction in which parallel rays of light coming from infinity are brought to focus in front of the retina due to having a long axial length or steep corneal curvature [1]. It is the leading cause of correctable visual impairment and preventable blindness worldwide [2]. The clinical feature of myopia includes reduction of distance vision, diminished color vision and contrast sensitivity [3].

Myopia is appearing with greater prevalence in young children [4, 5], which places these children at greater risk of developing high myopia. Myopia is more prevalent in Asian countries but relatively less common in Europe and North American countries [6]. Its prevalence rate in Africa is also lower as compared to Asian and European countries [6]. The prevalence was about 41% of the mid-to-late teenage population in the United States of America [7]. A study done in china among school students showed a prevalence rate of 36.71% [8]. In a study conducted in Taiwan school children, the prevalence rate of myopia ranges from 20% to 84% [9]. Whereas in a study done among black Africans children the prevalence rate reaches from 3% to 9.4% [10]. A study conducted in Gondar, Ethiopia among high school students showed a prevalence rate of myopia as 11.8% [2].

Myopia is the result of a combination of genetic and environmental factors [1, 6, 11], but the increases in myopia prevalence during the past century in certain societies argue strongly for a greater environmental inffuence [1]. It has been associated with age, sex, intensity of schooling, family history of myopia, time to outdoor activity, type of school, near working distance, and exposure to near work activities [2, 11–14]. As there is no universally accepted method for the prevention of myopia onset, it is important to identify modifiable risk factors associated with its development.

The worldwide increment in the prevalence of myopia has a large public health impact because of the associated risk in potentially blinding ocular conditions such as maculopathy, retinal detachment and optic neuropathy [5]. These could also impair vision-related quality of life and increase difficulty in performing vision-related tasks [15]. The economic costs of myopia are also high [6]. It has a considerable burden on individuals and society that can harm career choice, ocular health, and sometimes self-esteem [2, 8]. Public health interventions are essential to descend the growing myopia prevalence and its associated burden [7]. Blindness in children is among the priorities of WHO and the impact of blindness due to refractive errors including myopia is considered in terms of blind person-years [16]. Therefore, WHO recommends a regular visual screening program and provision of free spectacles for school children [17]. Ethiopia is one of the least developed countries in Africa, with relatively poor health service coverage especially of eye health care, and is believed to have one of the world’s highest rates of blindness [18].

There is limited study regarding the prevalence and associated factors of myopia among school children generally in Ethiopia, and particularly in the study area. So, the aim of this study was to assess the prevalence and associated factors of myopia among school children in Bahir Dar city, Northwest Ethiopia.

## Methods and materials

### Study design, setting, and period

School-based cross-sectional study was conducted in Bahir Dar city, Northwest Ethiopia, between October 18 and November 25, 2019. Bahir Dar is the capital city of the Amhara National Regional State, which is located 556 kilometers Northwest of Addis Ababa, the capital city of Ethiopia. The population of the city is estimated to be 389,177 in the year 2020 based on the 2007 Census [19]. Currently, the city has been divided into 6(six) sub-cities and 4 satellite towns found on the four directions of road exits. Satellite towns comprise 16% of the total population. According to the city Education office data, in the academic year of 2019/2020, there were 20(twenty) 1^st^ cycle primary schools, 55(fifty-five) 2^nd^ cycle primary schools, and 18(eighteen) secondary schools in the city. According to the city Health office report, the city comprising 2 government hospitals and 4 specialty private-owned clinics that provide eye care services including refraction. Currently, there is no government-owned spectacle dispensing unit in the city.

### Sample size determination

A total sample size of 634 was determined using a single population proportion formula by taking a prevalence of 50% since there is no similar study conducted in Ethiopia, by considering 95% confidence interval, 5% margin of error, design effect of 1.5 for multistage sampling, and 10% non-response rate.

### Sampling technique

A multistage sampling method was employed to select study participants from Bahir Dar city schools. Six schools; Shimbet, SOS, Fasilo, and Yekatit 12 from primary schools and Tana Haik and Bahir Dar Academy from secondary schools were selected by using a simple random sampling technique. Then by using proportional allocation for each school a systematic sampling technique was used to select each study participant.

### Source and study population

All children attending primary and secondary school in Bahir Dar city were the source population and those students attending the selected schools were the study population.

#### Inclusion and exclusion criteria

All Students aged 6–18 years old and attending school in Bahir Dar city were included in the study while those students with a history of recent ocular trauma and surgery were excluded from the study.

### Data collection tool and procedure

An interviewer-administered structured questionnaire was developed from different pieces of literature to assess the prevalence and risk factors of myopia. The questionnaires were completed by the parents or legal guardians of the children. We collected detailed information regarding socioeconomic status, parental education, parental history of myopia, working distance at near, distance from TV screen, time spent on using mobile, and outdoor activities. The questionnaire was pre-tested to check consistency in 5% of the participants in Gondar city and modification was considered according to its findings. The participants were recruited from Bahir Dar city schools and the examination of each child was conducted on their own respective school. Both interobserver and intraobserver agreement were determined between optometrists with respect to visual acuity measurement and refraction. Snellen acuity chart, Trial frame, Trial case, Pin-hole, Meter to measure the working distance, Retinoscopy, Direct Ophthalmoscope, and Torch were used for physical examination.

A data collection procedure involving two modes (administration of questionnaire and physical examination including refraction) was carried out. The data were collected by 3 senior optometrists and 2 ophthalmic nurses. The ophthalmic nurses interview the parents regarding socio-demographic data and they assess the visual acuity, finally, refraction and ophthalmoscopy were performed by the optometrists. Data collectors first introduce themselves and the purpose of the study. After they get consent from the subjects they proceeded for the vision examination and refraction. Visual acuity assessment was measured using a snellen chart. Visual acuity (VA) of every student was taken, and if the VA is less than 6/9 pinhole was used to identify whether the reduction of vision is due to refractive error or other ocular pathology. Those individuals with VA of less than 6/6 were refracted with dry retinoscopy and cycloplegic refraction. Cycloplegic refraction was performed for each student with 1% cyclopentolate eye drop, Two drops of cyclopentolate 1% were administered at least 30 min before refractive error measurement. Cycloplegia was considered complete if pupil dilated to 6 mm or more and there was no pupillary reflex. Retinoscopy was done using a streak retinoscope and subjective refraction was carried out to determine the final prescription of the students.

The information regarding the nature, effects, management, and prevention of myopia was given for all study subjects. Those who needed further examination were referred to Felege Hiwot Specialized Hospital secondary eye care center to get appropriate examination and management.

### Data quality control

The English version of questionnaire was translated to a similar form of Amharic version and back to English to increase the accuracy and consistency level of the questionnaire. To ensure the quality of data, a pretest was carried out on 5% of the sample size in Gondar city. Training had been given to data collectors and the supervisor for two days before data collection and the collected data were checked for completeness, accuracy, and clarity by the principal investigator daily during the data collection period. Then, the necessary correction has been given to the data collectors accordingly to the aim of the study.

### Data processing & analysis

The raw data were entered into EPI Info version 7. After data were coded and cleaned, it was exported to and analyzed by using SPSS version 22. Descriptive and summary statistics were performed to describe the study population by frequency, proportion, and summary statistics such as mean, standard deviation, and ranges. It was presented using tables. Both bi-variable and multivariable logistic regression analysis was done and variables with p-value < 0.2 under bi-variable logistic regression considered for multivariable logistic regression. In the multivariable logistic regression analysis, variables with a p-value of less than 0.05 were declared as statistically significant. Odds ratio with 95% confidence interval and the corresponding p-value was used to identify risk factors for the development of myopia.

### Operational definitions

#### Myopia

Defined as spherical equivalent refractive error (SER = sphere + 1/2 cylinder) of −0.50 D or more in at least one eye [2, 15, 20]. The degree of myopia < −3.00 D is categorized as low, −3.00 D to −6.00 D is labeled as moderate myopia, and if it is > −6.00 D it is categorized as high myopia.

#### Familial myopia

The presence of any degree of myopia in first-degree relatives diagnosed by eye care professionals.

#### Outdoor activity

Considered as yes if the child spent > 2 hour per day outdoor activities including playing game and sports.

#### Working distance

The regular distance at which a person adapts to do near tasks. The average working distance for a normal individual is 33 cm.

### Ethical clearance

Ethical clearance was obtained from the University of Gondar College of Medicine and Health Sciences and comprehensive specialized hospital, School of Medicine Ethical Review Committee in accordance with the declaration of Helsinki. Each participating school was visited a week before the data collection day, and permission to conduct the study was also obtained from the schools. A written informed consent form was given to each of the students aged 6–18 years to be taken to their parents or guardians the day before data collection. Students were only recruited if their parents or guardians gave assent and signed the consent forms, and were willing for the students to take part in the study. Verbal assent was also taken from the participants between the age ranges of 14 to 18 years old. Participants who were found to have myopia and any other ocular disorders were referred to Felege Hiwot Specialized Hospital secondary eye care center and underwent a full ocular examination. Participants with significant myopia were given their prescription to treat their optical disorders. Participant’s information was obtained with no identifier and confidentiality was maintained.

## Result

### Socio-demographic characteristics of the study participants

A total of 601 study participants were included in the study with a response rate of 94.8%. Thirty hundred thirteen (52.1%) of the study participants were male and half of them 301 (50.1%) were in the age category of 14–18 years. About 344 (57.2%) participants attend government school and 460 (76.5%) of the study participants were from primary school. Most of the study participants were Orthodox Christian, 510 (84.9%). One-third of the study participants’ parental educational status was found to be a primary school, 174 (29.0%), and half of the study participants 283 (47.1%) had a family monthly income of 2001–5000 ETB. Eighty-nine (14.8%) of the study participants had a familial history of myopia. More than half of the study participants 319 (53.1%) had mobile exposure of < 2 hour per day. Half of the study participants 299 (49.8%) had a near working distance of 33–60 cm and around 371 (61.7%) study participants had outdoor activity (Table 1).

**Table 1:** Socio-demographic characteristics of the study participants among school children of Bahir Dar city, Northwest Ethiopia, 2019 (n = 601).

Variable	Frequency	Percentage
Age Category		
6–9 years	250	41.6%
10–13 years	50	8.3%
14–18 years	301	50.1%
Sex		
Male	313	52.1%
Female	288	47.9%
School type		
Government	344	57.2%
Private	257	42.8%
Religion		
Orthodox	510	84.9%
Muslim	67	11.1%
Protestant	24	4.0%
Ethnicity		
Amhara	564	93.8%
Agew	16	2.7%
Tigrie	15	2.5%
Oromo	6	1.0%
Educational level of students		
Primary school	460	76.5%
Secondary school	141	23.5%
Parents educational level		
Unable to read and write	21	3.5%
Able to read and write	104	17.3%
Primary school	174	29.0%
Secondary school	158	26.3%
College and above	144	24.0%
Family monthly income		
< 2000 ETB	140	23.3%
2001–5000 ETB	283	47.1%
5001–10000 ETB	129	21.5%
> 10000 ETB	49	8.1%
Familial myopia		
Yes	89	14.8%
No	512	85.2%
Distance to television screen		
<2 meter	227	37.8%
2–4 meter	219	36.2%
>4 meter	156	26.0
Mobile exposure hours per day		
<2hrs/day	319	53.1%
2-4hrs/day	204	33.9%
>4hrs/day	78	13.0%
Working distance at near		
<33 cm	123	20.4%
33–60 cm	299	49.8%
>60 cm	179	29.8%
Outdoor activities		
No	230	38.3%
Yes	371	61.7%
Active rest during studying		
No	237	39.4%
Yes	364	60.6%

ETB = Ethiopian Birr, n = sample size.

### Proportion of myopia

Of the 601 students who participated in study 51 (8.49%) (95% CI: 6.20%, 10.80%) were myopic and among them, 31 (60.78%) had a moderate degree of myopia (Table 2).

**Table 2:** Proportion and degree of myopia among school children of Bahir Dar city, Northwest Ethiopia, 2019 (n = 601).

Variable	Frequency	Percentage
Myopia		
Yes	51	8.49%
No	550	91.5%
Degree of myopia (n = 51)		
Low	6	11.76%
Moderate	31	60.78%
High	14	27.46%

### Factors associated with myopia

The odds of being myopic among study participants within the age category of 10–13 years were 6.54 times more as compared to those within 6–9 years (adjusted odds ratio (AOR) = 6.54 (95% CI: 5.56, 10.69)). Whereas those participants within the age group of 14–18 years were 6.32 times more as compared to those within 6–9 years (adjusted odds ratio (AOR) = 6.32 (95% CI: 5.32, 9.69)).

The study participants who had 2–4 hours per day mobile exposure were 3.69 times more to develop myopia as compared to those who had < 2 hours per day exposure (AOR = 3.69 (95% CI: 1.63, 8.83)) whereas those who had > 4 hours per day mobile exposure were 11.6 times more to develop myopia as compared to those who had < 2 hours per day exposure (AOR = 11.6 (95% CI: 4.41, 30.42)). Similarly, the odds of being myopic among study participants who had a working distance of < 33 centimeters were 6.89 times more as compared to those participants who had a working distance of > 60 centimeters (AOR = 6.89 (95% CI: 2.71, 17.56)). The odds of being myopic among study participants who did not spend time for outdoor activities were 3.94 times more as compared to those who spent time in outdoor activities (AOR = 3.94 (95% CI: 1.87, 8.31)) (Table 3).

**Table 3:** Factors associated with myopia among school children, Bahir Dar city, Northwest Ethiopia, 2019 (n = 601).

Variables	Myopia		Crude odds ratio (95% CI)	Adjusted odds ratio (95% CI)
	Yes	No		
Sex				
Male	26	287	1.00	
Female	25	263	1.05(0.59–1.86)	
School type				
Government	20	324	1.00	1.00
Private	31	226	2.22(1.23–3.99)	1.18(0.57–2.41)
Age group(years)				
6–9	5	245	1.00	1.00
10–13	7	43	7.97(6.54–11.8)	6.54(5.56–10.86)*
14–18	39	262	7.29(5.93–11.5)	6.32(5.32–9.69)**
Family monthly income				
< 2000 ETB	10	130	1.00	
2001–5000 ETB	22	261	1.09(0.50–2.38)	
5001–10000 ETB	14	114	1.71(0.74–3.96)	
> 10000 ETB	4	45	1.15(0.34–3.87)	
Family history of myopia				
Yes	7	82	1.00	
No	44	468	1.10(0.48–2.53)	
Distance to television screen				
<2 meter	27	200	1.00	1.00
2–4 meter	17	201	0.63(0.33–1.18)	0.83(0.39–1.78)
>4 meter	7	149	0.35(0.15–0.82)	0.76(0.29–1.98)
Mobile exposure hours per day				
<2hrs/day	11	308	1.00	1.00
2-4hrs/day	23	181	3.56(1.69–7.47)	3.69(1.63–8.38)**
>4hrs/day	17	61	7.80(3.48–17.5)	11.6(4.41–30.42)***
Working distance at near				
<33 cm	29	94	5.65(2.65–12.8)	6.89(2.71–17.56)***
33–60 cm	13	286	0.86(0.36–2.05)	0.81(0.31–2.15)
>60 cm	9	170	1.00	1.00
Outdoor activities				
No	31	199	2.73(1.52–4.92)	3.94(1.87–8.31)***
Yes	20	351	1.00	1.00
Active rest during studying				
No	33	204	3.11(1.71–5.66)	1.21(0.22–6.81)
Yes	18	346	1.00	1.00

*p<0.05,

**p<0.01,

***p<0.001 cm = Centimeter, ETB = Ethiopian Birr, hrs/day = hours per day.

## Discussion

This study evaluated the prevalence of myopia and the associated risk factors among school children in Bahir Dar city, Northwest Ethiopia. The prevalence of myopia in this study was 8.49% (95% CI: 6.20%-10.80%). This finding is consistent with a study conducted in Welkite town, southwestern Ethiopia which was reported as 6.5% [20]. This might be due to the similarity in socioeconomic status and genetic factors among study participants. However, the prevalence of myopia in this study is higher as compared to studies done in Malaysia [21], Iran [22], Tanzania [13], and Debere Markose Ethiopia [23] which was reported as 5.4%, 4.35%, 5.6%, and 5.47% respectively. The differences could be attributed to the differentials in socioeconomic conditions, variations in the operational definitions, cut-off points of refractive errors, and factors related to environmental influences. Some studies from the above define myopia without spherical equivalent and also they used a cut-off point of > -1.00 Diopter whereas the present studies used a cut-off point of > -0.50 Diopter.

On the contrary, the result of this study is slightly lower as compared to studies done in Gondar, Ethiopia (11.9%) [2] and much lower than the studies conducted in America (41.5%) [7], Israeli (28.3%) [24], Taiwan (21%) [4], Beijing China (80.7%) [3], Hong Kong China (36.7%) [8], Delhi India (13.1%) [25], North India (21%) [14], Qingdao (22.6%) [26], Amman Jordan (17.6%) [27], and Saudi Arabia (17.2%) [28]. This difference might be because of the variation in race between the study participants and of Asian descent. Most Asian nations were more myopic as a result of a complex genetic trait and environmental factors responsible for myopia; and also in developed nations, students were subjected to excessive near tasks [2].

The odds of being myopic among study participants within the age category of 10–13 years and 14–18 years were 6.54 and 6.32 times more as compared to those within 6–9 years respectively. This result is supported by many studies conducted in America [7], Taiwan [4], China [8], India [14], Korea [29], Malaysia [21], Amman [27], Tanzania [13], and Debark and Kolla Diba, Ethiopia [30].

The study participants who had 2–4 and >4 hours per day mobile exposure were 3.69, 11.6 times more to develop myopia as compared to those who had < 2 hours per day exposure respectively. This finding is consistent with studies done in India [14, 25], Saudi Arabia [28], and Debere Markose, Ethiopia [23].

The odds of being myopic among study participants who had a working distance of < 33 cm were 6.89 times more as compared to those who had a working distance of > 60 cm. This result is supported by studies conducted in Singapore military conscripts [1], Sydney [11], China [3], and Gondar, Ethiopia [2]. This might be due to persistent short working distance leads to peripheral blur and inherent ciliary spasm that could cause myopia. However, the cross-sectional nature of the present study and the previous studies remained inconclusive whether a short reading distance was the cause or the consequence of myopia.

Similarly, the odds of being myopic among study participants who did not spend more time on outdoor activities were 3.94 times more as compared to those who spent > 2 hour per day in outdoor activities. It is in agreement with studies conducted in Sydney [11], India [25], Qingdao [26], Amman [27], and Gondar, Ethiopia [2]. The possible explanations might be that high ambient lighting could regulate the release of dopamine from the retina and stimulate the synthesis of vitamin D in the body [31, 32].

Positive parental history of myopia was a risk factor for the development of myopia in previous studies done in India [25], Saudi Arabia [28], Tanzania [13], and Gondar, Ethiopia [2]. But in this study family history was not significantly associated with myopia. This difference might be due to the variation in detecting myopia from the family. At present, there is no unified evidence about the prevalence of myopia among males or females. The current results revealed that females were not more likely to suffer from myopia than males. This is consistent with many previous studies done in different countries [2, 8, 25, 26, 30].

The strength of the present study was the way where refractometry was carried out. It was performed with cycloplegic refraction by applying cyclopentolate 1% eye drop for each study participant, which finally yields an accurate objective result. The limitation was since it was only a cross-sectional study; thus, we could not draw any conclusion about the risk factors of myopia.

## Conclusion

The prevalence of myopia was high among school children in Bahir Dar city. Older age, longer duration of mobile exposure and shorter near working distance were the risk factors for the development of myopia whereas having outdoor activity was identified as the protective factor of myopia. This study indicates the need for a regular visual screening program for school children. School screening program can help in early detection and management of myopia and prevents its associated complications. It is recommended those stakeholders or policy makers’ plan or design ways for direct school screening program.

## Supporting information

S1 Data (SAV)

S1 Annex English version of data extracting format. (DOCX)

## Abbreviations

AOR: Adjusted Odds Ratio
CI: Confidence Interval
COR: Crude Odds Ratio
EPI INFO: Epidemiological Information
ETB: Ethiopian Birr
SPSS: Statistical Package for Social Sciences
VA: Visual Acuity
WHO: World Health Organization
