# Supplementary material for: Prevalence and associated factors of myopia among school children in Bahir Dar city, Northwest Ethiopia, 2019
Source: PLoS One. 2021 Mar 22;16(3):e0248936. doi: 10.1371/journal.pone.0248936 (PMC7984635; doi:10.1371/journal.pone.0248936)
Supplement: S1 Annex — (DOCX) [file pone.0248936.s002.docx]

**S1 Annex. English version of data extracting format**

Pre tested structured questionnaire with data extracting format for prevalence and associated factors of myopia among school children Bahir Dar city, Northwest Ethiopia

**Introduction**

Good morning/afternoon, my name is --------------------------------. I am a member of a research group. I am studying the prevalence of myopia and associated factors among school children in Bahir Dar city by asking questions and doing ocular examination. Your appropriate answers for all of our questions are important to know prevalence of myopia and associated factors. Your answers will be confidential and keep in secret. If you decide that, you do not want to participate in the study now or at any time in the future; it is your right not to participate in the study. But we appreciate if you participate and will take 30 minutes for us to complete the questionnaire and the examination. Thank you. Next, I will read a consent, which assures your interest to participate.

Do I have your permission to continue?

If yes thank you and continue --------------

If no, thank you and go to next study subject --------------

Data collector

Name ----------------------- signature ------------------------ date -------------------

Checked by supervisor

Name --------------------- signature---------------------- date-------------------

| **Sociodemographic and family related variable** | | |
| --- | --- | --- |
| S.N | Questions | Response category |
|  | Age in years |  |
|  | Sex | 1. Male 2. Female |
|  | Religion | 1. Orthodox 2. Muslim 3. Protestant |
|  | Ethnicity | 1. Amhara 2. Agew 3. Tigrie 4. Oromo |
|  | Educational level of students | 1. Primary school 2. Secondary school |
|  | Parents educational level | 1. Unable to read and write 2. Able to read and write 3. Primary school 4. Secondary school 5. College and above |
|  | School type | 1. Government 2. Private |
|  | Family monthly income |  |
|  | Does anyone of your family have myopia? | 1. Yes 2. No |
|  | Do any one of your family wear spectacle for vision? | 1. Yes  2. No |
|  | If your answer is yes for Q 10 for what purpose he/she wear spectacle | 1. For distance  2. For near  3. For reading  4. For both distance and  near |
|  | Who wear spectacle among your family? | 1. Father  2. Mother  3. Brother/Sister |
|  | What is your usual working distance when you perform near tasks? | 1. < 33 cm 2. 33-55cm 3. > 60cm |
|  | What is your distance from television screen? | 1. <2 meter 2. 2-4 meter 3. >4 meter |
|  | Does the child had outdoor activities including game and sport activities? | 1. Yes 2. No |
|  | If yes to Question number 12, how many hours per day? | ………………………….. |
|  | Do you have active rest during studying( purposely looking far into the distance for 20 seconds every 20 minutes) | 1. Yes 2. No |
|  | What is your Mobile or other electronics exposure hours per day? | 1. < 2hrs/day 2. 2-4hrs/day 3. > 4hrs/day |

| **Checklist for ocular examination and refraction** | | | | |
| --- | --- | --- | --- | --- |
| S.N | Type of examination | OD | OS | OU |
|  | Visual acuity |  |  |  |
|  | Vision with pinhole |  |  |  |
|  | Dry objective refraction |  |  |  |
|  | Cycloplegic refraction |  |  |  |
|  | Subjective refraction |  |  |  |
|  | Visual acuity with Subjective refraction |  |  |  |
|  | Spherical equivalent refractive error |  |  |  |
|  | Any abnormal ocular finding findings |  |  |  |
